# The Mediating Role of Work Engagement Between Artificial Intelligence Anxiety and Task Performance Among Nurses: A Cross-Sectional Correlational Study

**DOI:** 10.3390/healthcare14162560

**Published:** 2026-08-16

**Authors:** Hamza Moafa

**Affiliations:** 1 Community and Psychiatric Mental Health Nursing Department, College of Nursing, King Saud University, Riyadh 11421, Saudi Arabia; hmoafa@ksu.edu.sa; 2 Family and Community Health Department, Dr. Sulaiman Al Habib College for Knowledge, Riyadh 13325, Saudi Arabia

**Keywords:** artificial intelligence anxiety, work engagement, task performance, nurses, mediation analysis

## Abstract

**Highlights:**

**What are the main findings?**

- Among nurses in Saudi Arabia, higher AI anxiety is positively associated with higher work engagement and higher task performance, and higher work engagement is positively associated with higher task performance.
- Work engagement shows a significant indirect (mediating) association between AI anxiety and task performance, suggesting a preliminary mediating pathway in this cross-sectional sample.

**What are the implications of the main findings?**

- Within the challenge–hindrance stressor framework, AI anxiety may be tentatively interpreted as a challenge rather than a hindrance stressor, with its positive association with task performance appearing to operate through work engagement.
- Strengthening AI utilization and competency, alongside supporting work engagement, may help nurses to better adapt to AI technologies, as moderate AI-related concern may be tentatively associated with favorable task performance.

**Abstract:**

**Background/Objectives:** As artificial intelligence (AI) continues to be integrated in healthcare settings, it enters clinical work as a support and a source of stress. Although AI anxiety acts as a psychological barrier to consistent engagement with AI technology, evidence on its relationships with nursing outcomes such as work engagement and task performance remains limited. This study examined the associations between AI anxiety, work engagement, and task performance among nurses using the challenge–hindrance stressor framework, considering work engagement as a hypothesized mediator. **Methods:** A quantitative cross-sectional correlational design was used. In December 2025, 106 nurses across various hospital units in Saudi Arabia were recruited through a non-probability strategy combining purposive, convenience, and snowball techniques. Participants completed the Artificial Intelligence Anxiety Scale, the 3-item Utrecht Work Engagement Scale, and the task performance subscale of the Individual Work Performance Questionnaire. Data were analyzed using Pearson correlations and PROCESS-based mediation analysis (Model 4), with 5000 bootstrap resamples and 95% percentile confidence intervals. **Results:** Work engagement statistically mediated the cross-sectional association between AI anxiety and task performance; the indirect effect was significant, whereas the direct effect was not. Higher AI anxiety was positively associated with higher work engagement (β=0.29, p=0.002), and higher work engagement with higher task performance (β=0.45, p<0.001). The total effect was significant (β=0.22, p=0.026), while the standardized indirect effect was 0.13 (95% CI: 0.036–0.248). **Conclusions:** AI anxiety may be tentatively interpreted as a possible challenge stressor, with its positive association with task performance appearing to operate indirectly through work engagement. These results indicate that strengthening AI competency and work engagement may support nurses’ task performance.

## 1. Introduction

Artificial intelligence (AI) is playing a critical role in driving technological transformation in multiple sectors [1], contributing to process optimization and intelligent decision making across organizations [2,3,4]. Recently, the integration of AI-based technologies into clinical settings (e.g., chronic disease management, clinical decision support, and predictive analytics) has added a new layer of complexity to the nursing work environment [5]. In this context, AI anxiety acts as a psychological barrier that limits consistent engagement with AI technology [6], manifesting as apprehension and avoidance behaviors linked to fear of operational mistakes and concerns about reduced professional relevance [7]. Accordingly, AI has received growing attention as a factor that may shape nursing practice.

Task performance, which refers to an individual’s ability to perform tasks and activities that are central to their job [8,9], is an essential outcome in nursing practice, with implications for organizational performance. Nurses perform many nursing and non-nursing tasks (NNTs), with the latter being associated with their efficiency and care quality. Previous research has indicated that nurses spend approximately one-third of their time performing NNTs [10], and these tasks have been negatively associated with the quality, productivity, and effectiveness of nursing care [11,12,13,14,15]. Therefore, identifying key factors associated with task performance among nurses is a priority.

Qualitative studies have provided valuable insights into nurses’ experiences with the integration of AI in healthcare, describing how they perceive, interpret, and use AI in practice [16,17,18,19,20]. Nurses have reported positive and negative attitudes toward AI applications. Most nurses acknowledged that AI enhanced their clinical judgment [16], and AI was viewed as a collaborative partner within healthcare teams that improved workflows and patient outcomes [20]. In one study, the potential for optimizing resource allocation and concerns about algorithmic bias and healthcare disparities were reported [19]. A qualitative synthesis of stakeholder beliefs concluded that AI use reduced workload and improved care [21]. In contrast, nurses have reported fears about job displacement and raised ethical concerns about data privacy [17], while other reports noted emotional disconnection in care and fears regarding the over-reliance on AI-driven systems among healthcare professionals, including nurses [18]. These qualitative approaches cannot test relationships or generate generalizable evidence, indicating a need for quantitative approaches. Quantitative research has documented associations between technology and positive job outcomes [22,23]; for example, technostress, when appraised as a challenge, was associated with work engagement and innovative behavior among employees of Chinese manufacturing firms [22], and direct positive and negative associations between challenge stressors and workplace performance have been reported [24]. Consequently, AI anxiety, understood here as an affective response to AI-related job demands, may be viewed through the lens of the challenge–hindrance stressor framework, even though anxiety is more often classified as a strain outcome than a stressor. Although quantitative studies exist, most rely on isolated regression models that do not reflect the complexity of theoretical frameworks, and mediation analysis may provide a more suitable approach for testing the mechanisms underlying this relationship.

Work engagement is a positive, fulfilling work-related state of mind characterized by vigor, dedication, and absorption [25], elements which capture the internal motivational forces that drive individuals toward targeted goals [25]. A recent study found that work engagement is moderately and positively associated with increased performance and quality of nursing activities [26]. These findings align with the job demands–resources (JD-R) model, which posits a positive association and proposes work engagement as a potential explanatory mechanism linking work demands to performance [27]. Work engagement was selected as the mediator because it represents the motivational mechanism through which job demands may translate into performance outcomes, as theorized by the JD-R model [27] and consistent with the motivational pathway posited by the CHSF [28]; empirical evidence also supports its association with nursing performance [26]. The present study is grounded in the challenge–hindrance stressor framework (CHSF), which has been used to explain workplace stressors and their consequences across a broad set of proximal and distal outcomes for over two decades [29]. The CHSF posits that job stressors are not uniformly detrimental but differ in their functional consequences depending on how individuals appraise them, distinguishing challenge stressors (job demands perceived as opportunities for growth, learning, and achievement) from hindrance stressors (perceived obstacles that constrain goal attainment and personal development) [28]. According to this framework, challenge and hindrance stressors are related to motivation and performance [28], and AI anxiety could, in principle, be perceived as a hindrance or challenge by nurses depending on their subjective appraisal.

To date, studies on AI in nursing have largely been qualitative or relied on isolated regression models, and quantitative evidence on nursing outcomes such as work engagement and task performance remains limited. As such, the specific mechanisms linking AI anxiety and task performance, particularly through work engagement, remain underexplored.

Therefore, the primary objective of this study was to examine the associations between AI anxiety, work engagement, and task performance among nurses using the challenge–hindrance stressor framework as an interpretive lens, considering work engagement as a hypothesized mediator. Based on the theoretical considerations and gaps in the literature discussed above, the following hypotheses were formulated:

1. AI anxiety is related to work engagement;
2. Work engagement is related to task performance;
3. AI anxiety is related to task performance;
4. Work engagement mediates the relationship between AI anxiety and task performance.

## 2. Materials and Methods

### 2.1. Design

This study employed a quantitative cross-sectional correlational design to examine the associations of AI anxiety with task performance and work engagement among nurses. The study was designed and documented following the STROBE checklist [30], which is provided as a Supplementary File (Supplementary Materials).

### 2.2. Participants, Setting, and Sample Size

In December 2025, nurses employed in patient care settings in Saudi Arabia were recruited through an online survey that they accessed from their personal devices. A non-probability sampling strategy was used, combining purposive, convenience, and refer-a-colleague (snowball) recruitment techniques. Eligible participants were nurses who (1) worked in a patient care setting and (2) reported using AI technology in patient care. Of 196 responses received, 106 valid responses were retained for analysis after excluding incomplete ones and those that did not meet the inclusion criteria. Specifically, 90 responses were excluded because respondents did not complete the survey in full or left the AI anxiety scale unfinished, precluding verification of the inclusion criteria and meaningful analysis.

The adequacy of the sample size was confirmed against recommendations from the relevant methodological literature [31,32]. Simulation studies employing the percentile bootstrap approach indicate that a minimum of approximately 90 participants provides adequate power (α=0.05; power =0.80) to detect a medium-sized indirect effect in a simple mediation model [31]. The final sample of 106 participants was therefore considered sufficient, as it exceeded this threshold.

### 2.3. Data Collection

Part of the data used in this study came from a broader research project, which the author had also drawn from in a previously published study [33].

The present study addresses a different research question, focusing on AI anxiety as a predictor of task performance and work engagement as a mediator within the challenge–hindrance stressor framework, with the analytic sample restricted to nurses who used AI technology in patient care. The analytic sample is a subset of the broader dataset used in [33], restricted to participants who completed the AI anxiety scale in full and met the inclusion criterion of using AI in patient care.

After ethical approval was obtained, the online survey was circulated through nurses’ networks to facilitate access to the intended participants. The survey included demographic data and three standardized scales; demographic and professional data included age, gender, educational level, and years of experience. After data collection was completed, responses were retrieved from Qualtrics (Provo, UT, USA) and prepared for statistical analysis.

### 2.4. Instruments

#### 2.4.1. Artificial Intelligence Anxiety Scale

AI anxiety was measured using the Artificial Intelligence Anxiety Scale (AIAS) [7]. This 21-item self-report instrument evaluates the respondent’s level of anxiety regarding AI technologies, with items rated on a 7-point Likert scale (1 = strongly disagree to 7 = strongly agree), yielding a total score range of 21 to 147; a higher score indicates higher levels of anxiety toward AI. The validity of the AIAS is supported by evidence of construct, criterion-related, and nomological validity, and it demonstrated excellent internal consistency (α=0.96) when previously applied to a nursing population [34].

The instrument was administered in its original English-language form without translation or adaptation. The Cronbach’s α for internal consistency in this study was 0.97. Consistent with the study’s research questions, which concerned overall AI anxiety rather than its specific dimensions, the total AIAS score was used; subscale-level analyses were reserved for future research.

#### 2.4.2. Utrecht Work Engagement Scale

Work engagement was measured using the 3-item version of the Utrecht Work Engagement Scale (UWES-3), an alternative to the longer version [35]. Each item was rated on a scale ranging from never (0) to always (6), with the total score calculated as the mean of the three items and a higher mean score indicating greater work engagement. The UWES-3 has demonstrated strong psychometric properties and validity, comparable with those of the longer version [35], and was chosen to reduce the burden on participants. The internal consistency reliability of the UWES-3 across five countries ranged from 0.77 to 0.85 [35], indicating acceptable reliability for an ultra-short measure. The Cronbach’s α of this subscale in this study was 0.85.

#### 2.4.3. Individual Work Performance Questionnaire

The Individual Work Performance Questionnaire (IWPQ) was developed by Koopmans et al. (2013, 2014) to measure work performance [8,36]. Only the task performance subscale was used, as the primary outcome of interest was nurses’ effectiveness in performing job-related tasks. This subscale consists of five items, each rated on a 5-point Likert scale ranging from seldom (1) to always (5), with a higher mean score indicating greater task performance. The internal consistency of the scale was previously confirmed, with a Cronbach’s α of 0.78 [36]. The Cronbach’s α of this subscale in this study was 0.93.

### 2.5. Data Analysis

All statistical analyses were conducted using the Statistical Package for the Social Sciences version 31 (SPSS; IBM Corp., Armonk, NY, USA). Statistical significance was defined as a two-tailed *p*-value less than 0.05. Participant characteristics and key study variables are summarized as frequencies, percentages, means, and standard deviations. Pearson correlation coefficients were used to examine the bivariate relationships between AI anxiety, work engagement, and task performance. A percentile bootstrapping method with 5000 resamples and 95% confidence intervals was used to estimate the indirect effect with Hayes’ PROCESS macro v4.2 (Model 4) [37]. The percentile bootstrap method does not assume normality of the sampling distribution of the indirect effect, and is therefore robust to potential non-normality in the study variables [37]. As the study variables were measured using different response metrics, completely standardized estimates are reported as the primary effect size index. Additionally, Harman’s single-factor test was conducted to assess common-method variance.

## 3. Results

### 3.1. Sociodemographic Information

Most of the nurses in the sample were female (n=82, 77.4%), and 34 (32.0%) were aged less than 30 years. Most participants held a Bachelor of Science in Nursing degree (n=64, 60.4%). The largest groups had more than 15 years of experience (n=31, 29.2%), worked in a general hospital (n=49, 46.2%), worked in the medical surgical ward (n=43, 40.6%), and worked rotating shifts (n=47, 44.3%). Full sociodemographic characteristics are presented in Table 1.

### 3.2. Scores on the Main Variables

On average, nurses scored 80.18±31.82 (out of 147) on the AIAS, 3.28±1.06 on the task performance subscale of the IWPQ, and 3.46±1.55 on the UWES-3 (Table 2).

The distribution of AI anxiety scores was approximately unimodal (skewness = 0.18, kurtosis = −0.31). Harman’s single-factor test indicated that one factor accounted for 44.96% of the variance, remaining below the 50% threshold and suggesting that common-method variance was unlikely to be a substantial concern.

### 3.3. Correlations Between AI Anxiety, Work Engagement, and Task Performance

Higher AI anxiety was positively correlated with higher task performance (r=0.216, p<0.05) and higher work engagement (r=0.294, p<0.01). Furthermore, higher work engagement was positively correlated with higher task performance (r=0.477, p<0.001) (Table 2).

### 3.4. Mediation Analysis

**Figure 1:**
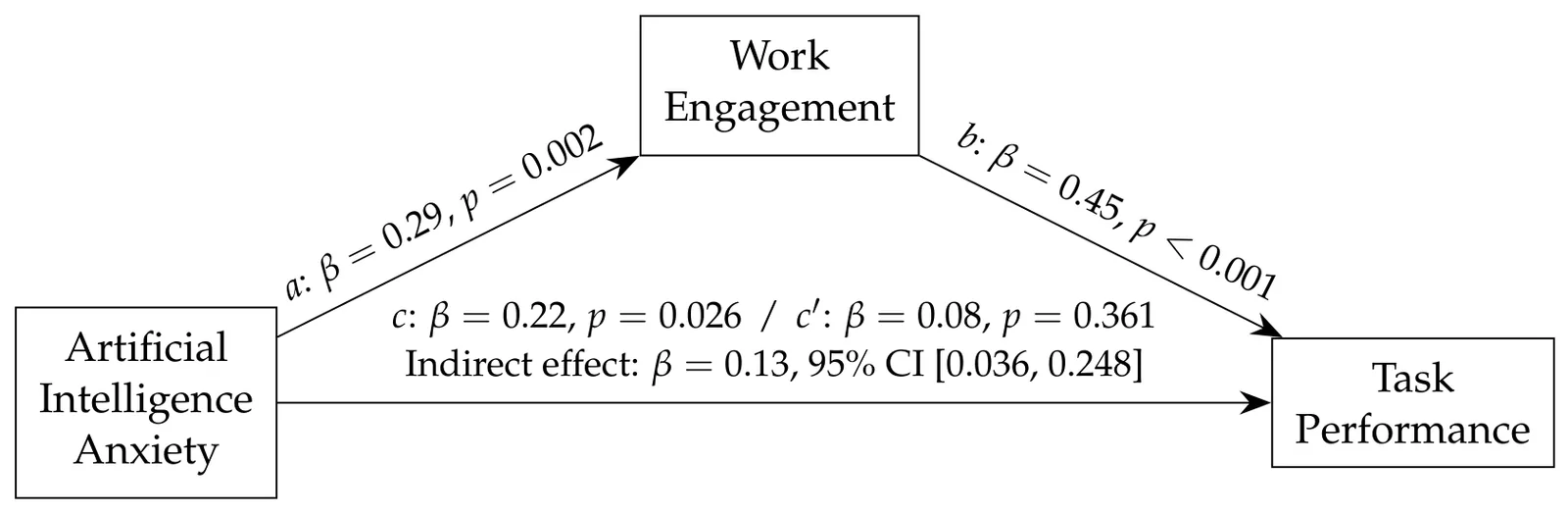
Mediation model with completely standardized path coefficients (N = 106). *a* = AI anxiety → work engagement; *b* = work engagement → task performance; *c* = total association; $c^{\prime }$ = direct association. $R^{2}$ for the mediator model = 0.087; $R^{2}$ for the outcome model = 0.234.

A simple mediation analysis was conducted to test whether work engagement mediated the relationship between AI anxiety and task performance, using the PROCESS macro for SPSS (Model 4) with 5000 bootstrap resamples and 95% percentile bootstrap confidence intervals (N = 106; Figure 1, Table 3). As the three measures were assessed using different response metrics, completely standardized estimates are reported as the primary effect size index.

Higher AI anxiety was positively associated with higher work engagement (path *a*: B=0.014, SE=0.005, 95% CI for *B*: [0.005, 0.024], t(104)=3.14, p=0.002; β=0.29), accounting for 8.7% of the variance (F(1,104)=9.87, p=0.002). Higher work engagement was positively associated with higher task performance after adjusting for AI anxiety (path *b*: B=0.309, SE=0.062, 95% CI for *B*: [0.187, 0.431], t(103)=5.02, p<0.001; β=0.45), with the model accounting for 23.4% of the variance in task performance (F(2,103)=15.74, p<0.001). The total association between AI anxiety and task performance was positive and significant (path *c*: B=0.007, SE=0.003, 95% CI for *B*: [0.001, 0.014], t(104)=2.26, p=0.026; β=0.22), whereas the direct association was not significant once work engagement was included in the model (path $c^{\prime }$: B=0.003, SE=0.003, 95% CI for *B*: [−0.003, 0.009], t(103)=0.92, p=0.361; β=0.08). The indirect association between AI anxiety and task performance through work engagement was significant, as its bootstrap confidence interval excluded zero (path a×b, completely standardized indirect effect =0.13, BootSE =0.054, 95% percentile bootstrap CI: 0.036–0.248). This pattern, in which the total association is significant but the direct association is not, is consistent with an indirect-only mediation effect [38].

## 4. Discussion

This study examined the hypothesized mediating role of work engagement in the relationship between AI anxiety and task performance among nurses. We found that work engagement statistically mediated this cross-sectional relationship, with a significant indirect effect and a non-significant direct effect. In particular, AI anxiety was positively and indirectly associated with task performance through work engagement, whereas the direct association was not significant once work engagement was considered. These findings are consistent with a tentative interpretation of AI anxiety, within the challenge–hindrance stressor framework, as a possible challenge-type stressor whose positive association with task performance appears to operate indirectly through work engagement; however, it should be noted that the challenge/hindrance appraisal was inferred from the direction of the observed associations, rather than measured directly.

The level of task performance in this study was slightly higher than that previously reported in a study conducted in Saudi Arabia [39] and in another from a different country [40]. The level of AI anxiety identified in this study was higher those reported among nurses in different countries [41,42,43,44] and was comparable with that reported among nursing students [45], whereas the work engagement level was slightly higher than that reported by Wu et al. [46]. Together, these findings suggest that AI anxiety, work engagement, and task performance vary with contextual conditions rather than being uniform across studies.

Our findings indicate that AI anxiety is positively associated with task performance in terms of the total association (path *c*), supporting Hypothesis 3. However, as the direct association (path $c^{\prime }$) was not significant once work engagement was included, this pattern is consistent with an indirect-only mediation effect [38], supporting the view of AI anxiety as a possible challenge stressor in this context. Work engagement was found to be moderately and positively associated with increased nursing productivity and quality of nursing activities [26], supporting Hypothesis 2. Meanwhile, AI anxiety was positively associated with work engagement, supporting Hypothesis 1. In contrast, among a non-nurse population, AI-induced stress was negatively associated with work engagement [47], highlighting that the positive association observed here may be context-specific and warrants replication.

Our results indicate that work engagement mediates the relationship between AI anxiety and task performance; in particular, the direct association between the two was no longer significant once work engagement was considered, supporting Hypothesis 4. This pattern is consistent with the hypothesized model informed by the CHSF, which suggests that AI anxiety may function as a challenge rather than a hindrance under certain conditions, such that its positive association with task performance appeared to operate indirectly through work engagement. However, the CHSF typically characterizes challenge stressors as demands appraised as growth opportunities, whereas anxiety is conventionally classified as a strain outcome. In this study, cognitive appraisal was not directly measured; accordingly, the challenge stressor interpretation is inferred from the positive directionality of the observed associations and should be treated as tentative. Our findings closely align with those of Ni et al. [48], who reported a positive association between job demands and job performance.

It should be noted that the magnitude of this indirect association was very small, suggesting that additional unmeasured factors may be associated with this relationship. The model accounted for only 23.4% of the variance in task performance, indicating that other unmeasured factors are likely important determinants of nursing task performance.

### Implications for Clinical and Assistive Practice

This study’s results have practical implications for nursing practice. Nurse managers should implement strategies to support task performance and work engagement, as the observed positive association between AI anxiety and task performance, operating indirectly through work engagement, tentatively suggests that moderate levels of AI-related concern may be associated with favorable performance outcomes rather than necessarily undermining them, at least in this cross-sectional sample. From a policy perspective, healthcare organizations should prioritize work engagement as a component of quality assurance; specific strategies may include structured AI training programs, phased implementation approaches, and peer support systems to help nurses adapt to AI technologies. Additionally, organizations should consider monitoring work engagement as an indicator of successful AI integration.

A key strength of this study is its grounding in well-established theoretical frameworks, which enhanced its conceptual clarity and supported the interpretation of the findings, particularly given the small effect size. The study also addressed a gap in the literature with an adequate sample size and validated instruments.

## 5. Limitations

This study had several limitations. First, the cross-sectional design precludes causal inferences; alternative directional orderings of the variables are statistically equally plausible, and the findings should be interpreted as associational patterns. The observed indirect effect was small (β=0.13, 95% CI: 0.036–0.248), and the findings should be treated as preliminary pending adequately powered replication.

Second, all variables were collected through a single self-report survey, which may have inflated associations through common-method variance. As an a priori design safeguard, participants were assured of anonymity and confidentiality during recruitment and data collection, and common-method variance was additionally assessed empirically. Consistent with the study’s research questions, which concerned overall AI anxiety, rather than its specific dimensions, the AIAS total score was used rather than its four subscales, and subscale-level analyzes were reserved for future research. Nonetheless, this choice may have masked differential associations between specific dimensions of AI anxiety and the outcome variables.

Third, the instruments were administered in their original English-language versions rather than in Arabic, considering that English is an established language of nursing education, clinical documentation, and professional practice in the study setting. Although a formal Arabic cross-cultural validation was beyond the scope of this study, it represents a worthwhile direction for future work.

Fourth, the non-probability sampling strategy may have limited the representation of nurses in different contexts and systematically favored nurses who were more comfortable with technology. The loss of 90 of 196 responses (45.9%) further compounds this concern, although the excluded responses were substantially incomplete, precluding comparative analysis. AI use was ascertained only through the self-reported eligibility criterion, and the specific type of AI used, such as clinical decision support systems, predictive analytics tools, AI-assisted documentation, or other applications, along with the frequency, duration, and voluntariness of use, was not assessed. As exposure is a likely effect modifier, this limits the ability to distinguish differential associations across varying levels of AI engagement. Cross-study comparisons should also be interpreted with caution, as differences in AI adoption rates, technological infrastructure, and cultural attitudes may account for the observed discrepancies.

Fifth, the mediation model was not adjusted for covariates, as including them in small-to-moderate samples can absorb meaningful variance and reduce power. The challenge or hindrance categorization was inferred from the direction of associations, rather than from direct measurement of cognitive appraisal. A supplementary quadratic AI anxiety term was non-significant for task performance (b=−0.0001, p=0.39) and work engagement (p=0.81), indicating no curvilinear association over the observed range. As a possible theoretical explanation, and consistent with the Yerkes–Dodson law [49], the sampled anxiety levels may not have reached the threshold beyond which performance would be expected to decline, such that only the ascending portion of the relationship was captured and no curvature was detectable in the utilized data.

Future research should employ longitudinal designs with larger, probability-based samples; incorporate covariate adjustment, subscale-level analyses, cognitive appraisal measures, and formal common method bias assessments; and operationalize AI exposure more precisely.

## 6. Conclusions

This study provides an initial empirical examination of the associations between AI anxiety, work engagement, and task performance among nurses, and is among the first to test a mediated pathway linking AI anxiety and task performance through work engagement. The observed indirect effect is consistent with the theoretical relevance of this proposed mechanism, whereas the small effect size reflects the likelihood that task performance is determined by multiple factors. Given the cross-sectional study design and the tentative nature of the challenge stressor interpretation, these findings should be considered preliminary and require replication with stronger designs. Work engagement should therefore be viewed as a component of broader intervention strategies aimed at supporting nurses’ adaptation to AI technologies. 

## Figures and Tables

**Table 1:** Sociodemographic characteristics of the participants (N = 106).

Variable	Category	*n*	%
Age (years)	<30	34	32.0
	30–34	15	14.2
	35–39	30	28.3
	40–44	21	19.8
	45–49	4	3.8
	≥50	2	1.9
Gender	Female	82	77.4
	Male	24	22.6
Level of education	Diploma	26	24.5
	Bachelor	64	60.4
	Masters	16	15.1
Years of experience (years)	<1	6	5.7
	1–5	28	26.4
	6–10	17	16.0
	11–15	24	22.6
	>15	31	29.2
Current job position	Staff nurse	75	70.8
	Charge nurse	10	9.4
	Nurse manager	7	6.6
	Clinical instructor	3	2.8
	Faculty member	1	0.9
	Other	10	9.4
Department/unit	Medical surgical	43	40.6
	Emergency	12	11.3
	Intensive care unit	3	2.8
	Maternity/obstetrics	1	0.9
	Pediatric	8	7.5
	Outpatient clinic	9	8.5
	Other	30	28.3
Healthcare facility type	Primary health center	20	18.9
	General hospital	49	46.2
	Specialized hospital	18	17.0
	University/academic institution	9	8.5
	Private hospital or clinic	2	1.9
	Other	8	7.5
Nationality	Saudi	78	73.6
	Non-Saudi	28	26.4
Usual work shift	Morning shift	45	42.5
	Evening shift	3	2.8
	Night shift	7	6.6
	Rotating shifts	47	44.3
	Other	4	3.8

**Table 2:** Means, standard deviations, and Pearson correlations between study variables (N = 106).

Variable	Min	Max	M	SD	1	2	3
1. AI anxiety	21	147	80.18	31.82	—		
2. Task performance	1	5	3.28	1.06	0.216 *	—	
3. Work engagement	0	6	3.46	1.55	0.294 **	0.477 ***	—

Note: AI = artificial intelligence; * p<0.05, ** p<0.01, *** p<0.001.

**Table 3:** Direct and indirect associations between AI anxiety, work engagement, and task performance (N = 106).

Path	B	SE	β	*p*	Boot SE	95% CI [LL, UL]
Path *a* (AI anxiety → work engagement)	0.014	0.005	0.29	0.002	—	[0.005, 0.024]
Path *b* (work engagement → task performance)	0.309	0.062	0.45	<0.001	—	[0.187, 0.431]
Total effect (*c*)	0.007	0.003	0.22	0.026	—	[0.001, 0.014]
Direct effect ($c^{\prime }$)	0.003	0.003	0.08	0.361	—	[−0.003, 0.009]
Indirect effect	—	—	0.13	—	0.054	[0.036, 0.248]

Note: N = 106. AI = artificial intelligence. Estimates were generated using the PROCESS macro for SPSS (Model 4) with 5000 bootstrap samples. B = unstandardized coefficient; β = completely standardized coefficient. The indirect effect is reported as the completely standardized indirect effect (β). Boot SE = bootstrap standard error based on 5000 bootstrap samples. The 95% CIs for paths *a*, *b*, the total effect, and the direct effect are ordinary confidence intervals for the unstandardized coefficients (*B*) from the PROCESS output; the 95% CI for the indirect effect is the percentile bootstrap confidence interval for the completely standardized indirect effect. The indirect effect is evaluated by whether its CI excludes zero, rather than by a *p*-value. The small magnitude of the unstandardized coefficients involving AI anxiety reflects the wide AIAS scoring range (21–147); the standardized coefficients (β) provide a scale-free interpretation. $R^{2}$ for the mediator model (path *a*) = 0.087; $R^{2}$ for the outcome model (paths *b* and $c^{\prime }$) = 0.234. — = not applicable.

## Data Availability

The datasets generated during the current study are not publicly available owing to the personal nature of the topic; however, they may be made available from the corresponding author upon reasonable request. The analytic sample in the present study is a subset of the broader dataset used in a previously published study [33], which examined a different research question, predictor, and theoretical framework.

## Supplementary Materials

The following supporting information can be downloaded at: https://www.mdpi.com/article/10.3390/healthcare14162560/s1, STROBE Statement—Checklist of items that should be included in reports of cross-sectional studies.

## Abbreviations

The following abbreviations are used in this manuscript:

AI	Artificial intelligence
AIAS	Artificial Intelligence Anxiety Scale
CHSF	Challenge–hindrance stressor framework
IWPQ	Individual Work Performance Questionnaire
JD-R	Job demands–resources
NNTs	Non-nursing tasks
UWES-3	Utrecht Work Engagement Scale (3-item)
